# Prevalence of insufficient outdoor activity and caregiver-related correlates among school-aged children in Beijing, China: a cross-sectional analysis

**DOI:** 10.3389/fpubh.2026.1816425

**Published:** 2026-05-26

**Authors:** Liyu Huang, Lulu Meng, Yiran Li, Siyu Liang, Wenjia Li, Manning Wang, Yan Zhang, Jiali Duan, Ruoxiang Cao

**Affiliations:** 1 Institute of Nutrition and Food Hygiene, Beijing Center for Disease Prevention and Control, Beijing, China; 2 School of Public Health, Capital Medical University, Beijing, China; 3 Office of Research and Teaching Administration, Beijing Center for Disease Prevention and Control, Beijing, China; 4 School of Public Health, Hebei Medical University, Shijiazhuang, Hebei, China; 5 Xingtai Xindu District Center for Disease Control and Prevention, Xingtai, Hebei, China

**Keywords:** caregivers, outdoor activity, prevalence, school-aged children, time outdoors

## Abstract

**Background:**

Daily outdoor activity time among school-age children worldwide is on a downward trend. This study updated the daily outdoor activity status of Chinese school-age children and analyzed the factors associated with insufficient outdoor activity among school-age children.

**Methods:**

This study utilized baseline data from a sugar-sweetened beverage consumption behavior cohort conducted among primary and secondary schools in Beijing, China. The baseline survey was conducted from October to November 2024, and 3,370 school-age children with complete data related to outdoor activity were included in the analysis. Binary logistic regression model was used to analyze the main factors associated with insufficient outdoor activity among school-age children.

**Results:**

The overall prevalence of insufficient outdoor activity was 82.9% among Beijing school-aged children (< 2 h/day).Residential location, grade, weight status, pre-activity beverage consumption patterns, and caregivers' daily outdoor activity duration were closely correlated with inadequate outdoor activity, with notable heterogeneity observed across residential area, gender and grade subgroups. Caregivers' limited outdoor activity participation emerged as the most stable correlate across all stratifications.

**Conclusion:**

This study reveals a high prevalence of insufficient outdoor activity among Beijing schoolchildren and identifies distinct population-specific associated factors. The findings provide empirical evidence for developing targeted subgroup interventions and optimizing family–school collaborative strategies to improve outdoor activity levels and promote long-term health among urban school-aged children.

## 1 Introduction

Outdoor activity is commonly defined as physical activities conducted outdoors during daylight hours (from sunrise to sunset) with exposure to natural light, including outdoor physical education classes and extracurricular sports activities, walking, cycling, and other recreational activities (1–3). Outdoor activity during daylight hours is consistently associated with improved health outcomes in school-aged children, most notably reduced myopia risk, as well as benefits for immune function, skeletal development, obesity prevention, and psychological wellbeing (1–10). Current guidelines, including Chinese national recommendations, advise a minimum of 2 h of daily outdoor activity for children, a benchmark widely adopted to support long term health (1, 11–14).

Global trends indicate a concerning decline in outdoor activity among children, with estimates showing 81.0% of school-aged children worldwide fail to meet physical activity recommendations (15). In China, national and regional surveys have documented similarly low adherence: a 2016 national study found 74.3% of children had less than 2 h of daily outdoor activity, while data from Beijing in 2019 and 2022 reported proportions of 71.9% and 65%, respectively (16, 17). These trends are likely driven by shifting lifestyles, including increased screen time and urbanization (18–20), yet factors shaping outdoor activity patterns in large Chinese cities remain understudied.

As a country with a large number of school-age children, China has approximately 203.22 million school-age children aged 6–17, a figure calculated based on data from the Seventh National Population Census (21). In China's education system in 2023, there were approximately over 200 million school-age children aged 6–17 at school, with a school attendance rate of 92%, ranking among the top in the world (22). Beijing, as a major urban center, offers a critical context for understanding these patterns, with over 1.81 million school-aged children enrolled in its primary and secondary schools (23). Characterizing outdoor activity and its correlates in this population is essential to inform targeted public health strategies. The study uses cross-sectional baseline data from a 2024–2025 cohort study conducted in Beijing primary and secondary schools to estimate the prevalence of insufficient daily outdoor activity and examine its correlates. Specifically, this study aims to explore whether residential area, gender, grade level, weight status, multi-stage beverage consumption behaviors, and caregiver characteristics (including educational level and daily outdoor activity duration) are associated with insufficient outdoor activity. The findings will provide evidence to inform context-specific interventions aimed at promoting healthy outdoor activity levels among urban school-aged children.

## 2 Materials and methods

### 2.1 Study design and study population

This study was a cross-sectional analysis using baseline data from a prospective cohort study investigating sugar-sweetened beverage consumption and related health behaviors among school-aged students in Beijing, China. Baseline data were collected between October and November 2024 across three districts: Dongcheng (central urban), Fengtai (outer urban), and Daxing (suburban). A multi-stage stratified cluster sampling strategy was applied. Districts were categorized into three strata based on population density, geographic location, and socioeconomic development: central urban, outer Urban, and suburban areas. Participants were selected by inclusion criteria: (1) Currently enrolled in primary, middle, or high school in Beijing; (2) Student and primary caregiver provided written informed consent; (3) Able to understand and complete study questionnaires independently or with assistance. Exclusion criteria: (1) On prolonged sick leave or absent during survey; (2) Unable to comprehend or cooperate with data collection; (3) Declined to participate. Of the 3,514 students who participated the baseline survey, 3,370 were included in the final completed case analysis.

Sample size calculation. Sample size was calculated based on a 2022 local estimate of insufficient outdoor activity ( ≤ 2 h/day) among Beijing school-age children (*p* = 0.65) (17). The required sample size was computed using the formula *N* = $\mu_{\text{α}/2}^{2}\times$*p*×(1-*p*)/δ^2^×*deff*, where μ_α/2_ = 1.96 for a 95% two-sided confidence level, the margin of error δ = 0.15 × *p* = 0.0975, and the design effect deff = 2. A 10% non-response or invalid rate was incorporated. The minimal required sample per stratum was 205, yielding a total minimal sample of 615 for three strata. The final analytic sample of 3,370 provided ample statistical power and precision.

Data were collected using a mixed approach of interviewer-administered and self-administered questionnaires. For primary school students, interviews were primarily conducted face-to-face by investigators at school. For middle and high school students, surveys were mainly self-administered.

The study was approved by the Ethics Committee of the Beijing Center for Disease Prevention and Control (BJCDC2024031). Written informed consent was obtained from the students' caregivers and from the participating students prior to the study.

### 2.2 Data collection

Two self-designed questionnaires were used in the survey: a sugar-sweetened beverage consumption behavior questionnaire for school-aged children and a family environment questionnaire. These questionnaires were developed based on the Chinese Dietary Guidelines (2022), the Encyclopedia of Nutrition Science, and other literature (2, 3, 13, 24), and refined through focused group discussions, expert review, and repeated pretesting. Invited experts with rich experience and professional knowledge in related fields, including epidemiologists, public health experts, nutritionists, and school health education teachers, to review and evaluate the questionnaire. Based on the experts' revision suggestions, ensure that the questionnaire has good content validity and scientificity. The Cronbach's alpha coefficients were 0.81 and 0.75, respectively, indicating good reliability. The questionnaires mainly covered basic demographic information, beverage consumption-related information, outdoor activity, and other health-related behavioral information. The sugar-sweetened beverage consumption behavior questionnaire and the family environment questionnaire were completed independently by the students and their parents, respectively. Student questionnaires were administered in school under teacher supervision, while caregivers questionnaires were completed at home. The responses from students and caregivers were matched one-to-one via questionnaire codes.

#### 2.2.1 Demographic information

Demographic characteristics included residential area (central urban, outer urban, suburban), gender, and grade (primary, middle, high school).

#### 2.2.2 Outdoor activity assessment

Daily daytime outdoor activity in the past week was assessed by a single self-report item: In the past week, what was your average daily daytime outdoor activity duration? Response were categorized as: < 0.5 h, 0.5–1 h, 1–2 h, ≥2 h. Outdoor activity was defined as physical activity performed outdoors, including outdoor physical education, extracurricular sports, walking, cycling, and other related activities. For analytical purposes, sufficient outdoor activity was defined as ≥2 h per day, and insufficient outdoor activity as < 2 h per day.

Given the self-reported and retrospective nature of this measure, responses are considered categorical estimates rather than precise quantitative values. To reduce recall bias, we employed the following controls: (1) the definition of outdoor activity (including outdoor physical education, extracurricular sports, walking, cycling, etc.) was standardized within the questionnaire to minimize misinterpretation; (2) recall was anchored to the average daily duration of daytime outdoor activity over the most recent week, balancing cognitive load and representativeness; (3) recall was tied to concrete daily events (e.g., school days, after-school activities, weekend outdoor plans) to help respondents recall activity within specified time periods.

#### 2.2.3 Weight status assessment

Height and weight were measured by trained school health professionals. According to the People's Republic of China industry standard Anthropometric measurements method in health surveillance (WS/T 424-2013) (25). The data were obtained from the routine physical examination records of the school. Body mass index (BMI) was calculated as weight (kg) divided by height squared (m^2^).According to the health industry standard of the People's Republic of China of China industry standard Screening standard for malnutrition of school-age children and adolescents (WS/T 456-2014), wasting is acute malnutrition caused by insufficient current dietary protein–energy intake, which results in a BMI below the age- and sex-specific BMI reference cutoffs and thus can be diagnosed as wasting (26).According to the standard Screening for overweight and obesity among school-age children and adolescents (WS/T 586-2018), using the age- and sex-specific BMI cutoff points as showed in Supplementary Table 1,Overweight was diagnosed when BMI was ≥ the overweight cutoff but < the obesity cutoff for the corresponding sex and age group, and obesity was diagnosed when BMI was ≥ the obesity cutoff for the corresponding sex and age group (27).This standard is applicable to the screening of overweight and obesity among school-age children of all ethnic groups in all regions of China. It was issued and promoted for use by the National Health Commission of the People's Republic of China.

#### 2.2.4 Beverage consumption before/during/after outdoor activities

Beverage consumption before, during, and after outdoor activity was assessed by three parallel items: “What did you drink before/during/after outdoor activities?” Responses: nothing; only plain water; occasional ( ≤ 1/week); sometimes (2–3/week); often (≥4/week).

#### 2.2.5 Family-related factors

In this study, family-related factors included the number of children in the family (≥2 or 1), the educational level of the caregivers (high school or below, college or above), and caregiver's daily outdoor activity duration (same categories as children).

### 2.3 Quality control

A three-level quality control system was established at the project team, project sites, and survey schools. Investigators underwent standardized training and were deemed qualified upon assessment. During field surveys, procedures were strictly carried out in accordance with the survey protocol. On-site quality control personnel promptly identified and corrected omissions, logical errors, and other issues detected in the field. After the survey, data were entered into a database using EpiData 3.1 by professionally trained personnel, with double data entry used to input, verify, and clean the survey data.

### 2.4 Statistical analysis

Statistical analyses were performed using SPSS version 21.0. Categorical data were presented as counts (*n*) and percentages (%). The distribution of outdoor activity duration among school-age children was categorized as (< 0.5 h/day, 0.5–1 h/day, 1–2 h/day, ≥2 h/day). Group differences in the distribution of daily outdoor activity duration were examined using the chi-square test. The univariate analysis of the prevalence of insufficient outdoor activity were assessed using the chi-square test. As the sample size was sufficiently large and only 144 participants had missing data (equivalent to 4.1% of the total), missing data were not imputed, and all statistical analyses were performed using complete cases only.

Multivariable binary logistic regression was conducted to examine adjusted cross-sectional associations with insufficient outdoor activity. Adjusted models included *a priori* confounders: residential area, gender, grade, weight type status, beverage consumption, number of children in family, caregiver education, and caregiver outdoor activity duration. The dependent variable was coded as insufficient outdoor activity (1 = < 2 h/day; 0 = ≥2 h/day). All models were adjusted for *a priori* confounders including residential area, gender, grade, ethnicity, household registration type, weight status, beverage consumption patterns, family structure, caregiver education, and caregiver daily outdoor activity time. The independent variable coding is as follows: Residential area (central urban = 0; outer urban = 1; suburban = 2), Gender (girls = 0; boys = 1), Grade (middle school = 0; primary school = 1; high school = 2), Ethnicity (han = 0; others = 1), Household registration type (agricultural = 0; urban = 1), Weight type status (normal = 0; underweight = 1; overweight = 2; obesity = 3), Beverage consumption type before outdoor activity (nothing = 0; only plain water = 1; occasionally beverages = 2; sometimes beverages = 3; often beverages = 4), Beverage type during outdoor activity (nothing = 0; only plain water = 1; occasionally beverages = 2; sometimes beverages = 3; often beverages = 4), Beverage type after outdoor activity (nothing = 0; only plain water = 1; occasionally beverages = 2; sometimes beverages = 3; often beverages = 4), Number of children in family (≥2 children = 0; 1 child = 1), Caregiver's education (high school or below = 0; college or above = 1), Caregivers' daily outdoor activity duration (< 0.5 h/day = 0; 0.5–1 h/day = 1; 1–2 h/day = 2; ≥2 h/day = 3).

Subgroup analyses were conducted by residential area (central urban, outer urban, suburban), gender (boys, girls), and grade (primary, middle, high school) to further characterize patterns associated with insufficient outdoor activity among school-aged children. Each stratification variable was excluded from the corresponding subgroup model, with all other model specifications kept identical to those used in the primary analysis. All statistical significance tests were two-sided, and a level of *p* < 0.05 was considered statistically significant.

## 3 Results

### 3.1 Baseline characteristics

**Figure 1:**
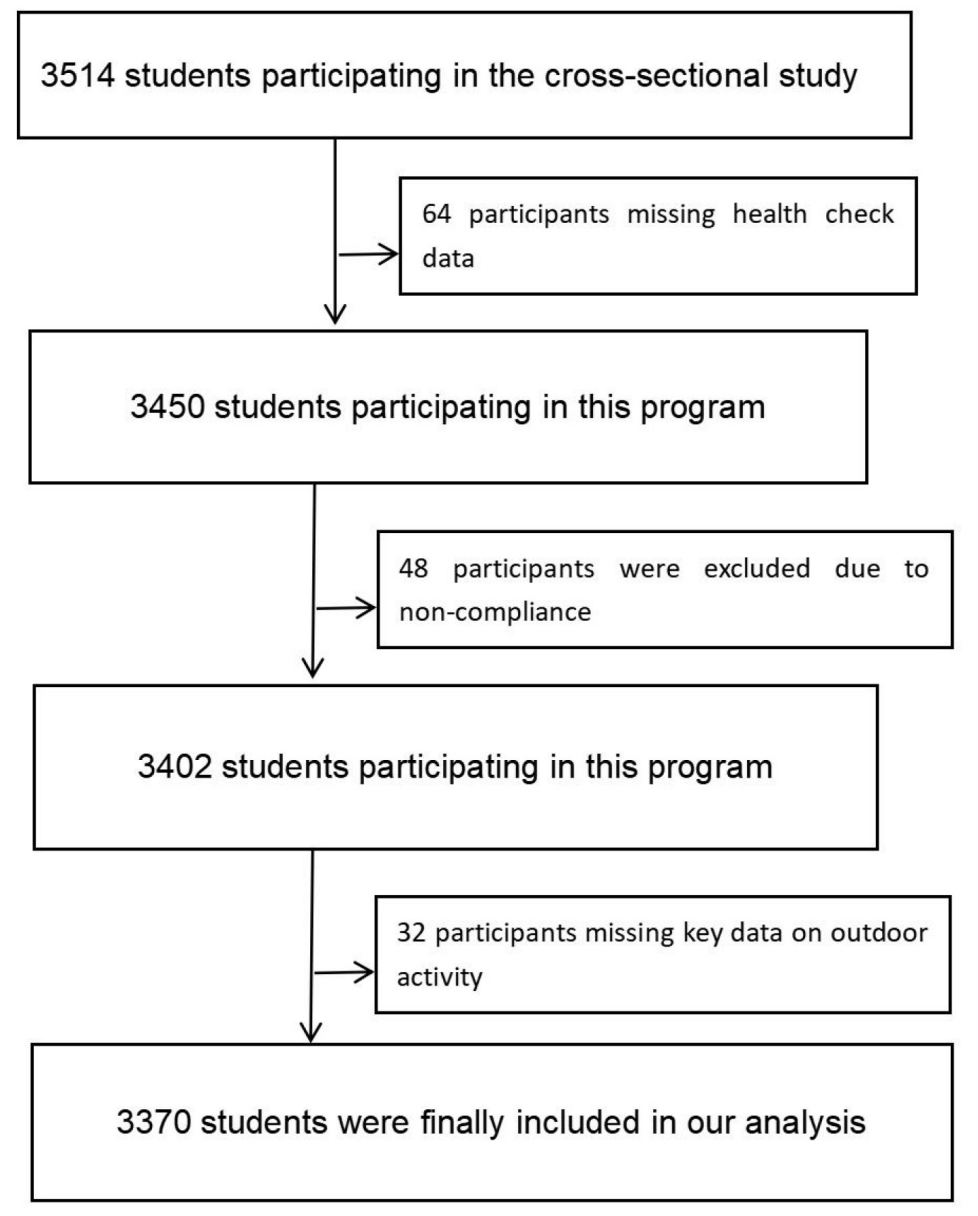
Flow diagram of study participants.

Participant flow is shown in Figure 1. Of the 3,514 school-age children enrolled in the cross-sectional study, 64 were excluded due to missing health check data, leaving 3,450 eligible participants. An additional 48 were excluded for non-compliance, resulting in 3,402 participants. A final 32 were excluded for missing key data on outdoor activity, yielding an analytic sample of 3,370 students. The overall effective inclusion rate was 95.9%.

Baseline characteristics of the study population are presented in Table 1. Of 3,370 participants, 34.7% of the school-age children were from central urban area, 31.9% from outer urban area, and 33.4% from suburban area. Boys accounted for 50.6% and girls for 49.4%.Primary, middle, and high school students accounted for 31.1%, 34.8%, and 34.1%, respectively. The majority were of Han ethnicity (91.4%), while 8.6% were of the other ethnicities. The majority were of urban household registration (88.6%), while 11.4% were of agricultural household registration. Beverage consumption patterns surrounding outdoor activity were as follows. Before outdoor activity, 16.5% consumed nothing, 52.0% drank only plain water, 23.8% consumed beverages occasionally, 6.0% sometimes, and 1.7% often. During outdoor activity, 24.5% consumed nothing, 44.1% drank only plain water, 20.0% occasionally consumed beverages, 8.9% sometimes, and 2.5% often. After outdoor activity, 7.2% consumed nothing, 46.5% drank only plain water, 29.7% occasionally consumed beverages, 12.8% sometimes, and 3.9% often. The proportion of students from families with one child was 60.9%, and the proportion from families with two or more children was 39.1%, 37.3% caregivers had high school or below, and 62.7% had college or above. Caregiver daily outdoor activity duration was distributed as follows: < 0.5 h (22.5%), 0.5–1 h (42.9%), 1–2 h (23.8%), and ≥2 h (10.8%).

**Table 1:** Characteristics of the participants.

Characteristics	Variables	Frequency (percentage) *n* (%)
Total		3,370 (100.0)
Residential area	Central urban	1,170 (34.7)
	Outer urban	1,076 (31.9)
	Suburban	1,124 (33.4)
Gender	Boys	1,705 (50.6)
	Girls	1,665 (49.4)
Grade	Primary school	1,049 (31.1)
	Middle school	1,171 (34.8)
	High school	1,150 (34.1)
Ethnicity	Han	3,081 (91.4)
	Others	289 (8.6)
Household registration type	Agricultural	383 (11.4)
	Urban	2,987 (88.6)
Weight type status	Normal	1,863 (55.3)
	Underweight	214 (6.4)
	Overweight	603 (17.9)
	Obese	690 (20.5)
Beverage consumption before outdoor activities	Nothing	558 (16.5)
	Only plain water	1,753 (52.0)
	Occasional beverage	802 (23.8)
	Sometimes beverage	201 (6.0)
	Often beverage	56 (1.7)
Beverage consumption during outdoor activities	Nothing	824 (24.5)
	Only plain water	1,487 (44.1)
	Occasional beverage	675 (20.0)
	Sometimes beverage	300 (8.9)
	Often beverage	84 (2.5)
Beverage consumption after outdoor activities	Nothing	241 (7.2)
	Only plain water	1,566 (46.5)
	Occasional beverage	1,002 (29.7)
	Sometimes beverage	431 (12.8)
	Often beverage	130 (3.9)
Number of children in family	≥2	1,319 (39.1)
	1	2,051 (60.9)
Caregivers' educational	High school or below	1,258 (37.3)
	College or above	2,112 (62.7)
Caregivers' daily outdoor activity duration	< 0.5 h/day	758 (22.5)
	0.5–1 h/day	1,447 (42.9)
	1–2 h/day	801 (23.8)
	≥2 h/day	364 (10.8)

### 3.2 Distribution of outdoor activity duration

**Figure 2:**
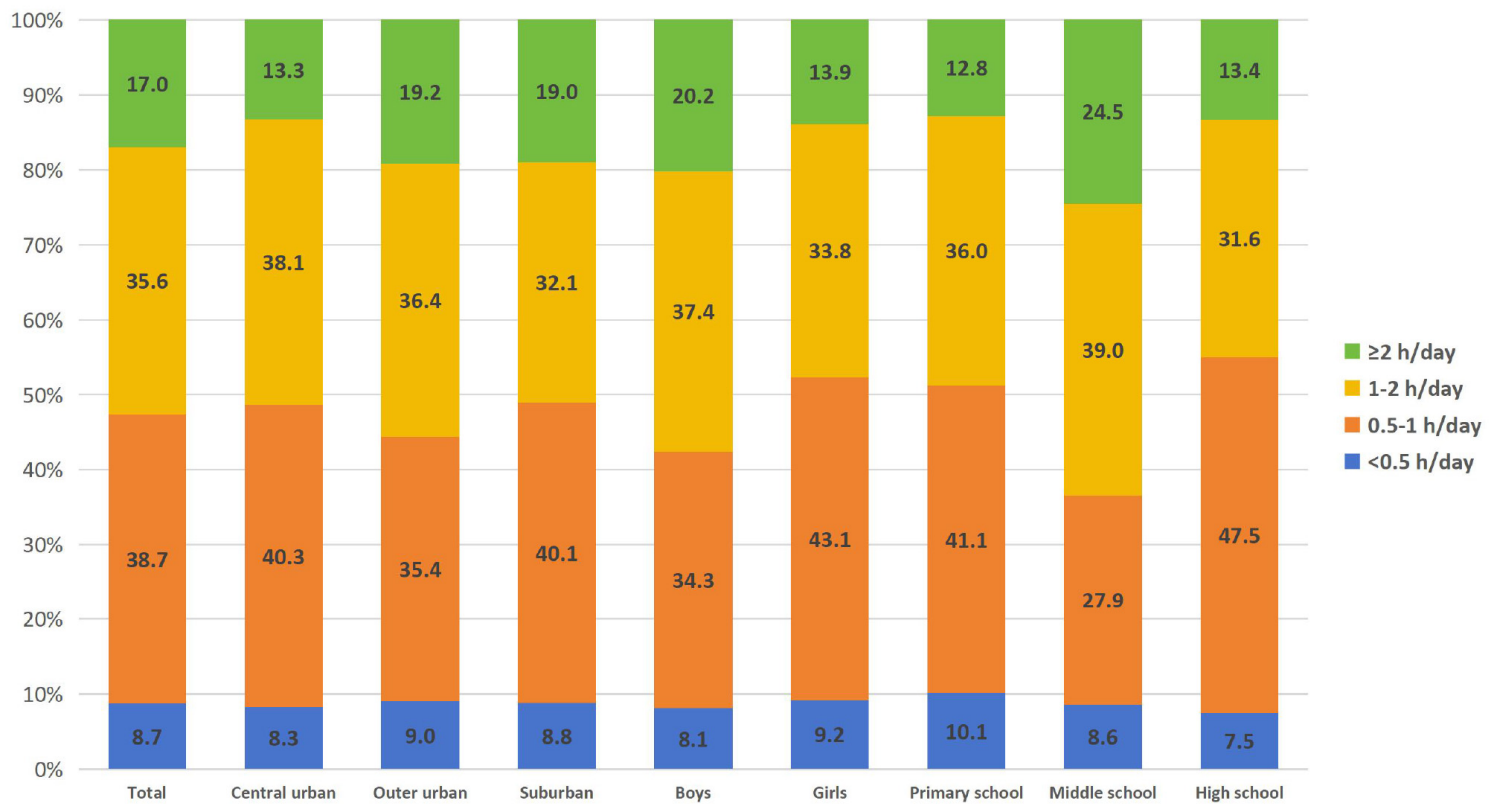
Distribution of daily outdoor activity duration of school-age children in different residential area, gender and grade.

As illustrated in Figure 2, the distribution of daily outdoor activity duration was: < 0.5 h (8.7%), 0.5–1 h (38.7%), 1–2 h (35.6%), and ≥2 h (17.1%). Differences in the distribution of outdoor activity duration among groups defined by residential area (**χ**^**2**^ = 25.455, *p* < 0.001), gender (**χ**^**2**^ = 40.773, *p* < 0.001) and grade (**χ**^**2**^ = 131.953,*p* < 0.001) were statistically significant.

### 3.3 Prevalence of insufficient outdoor activity

Table 2 shows the univariate analysis of the prevalence of insufficient outdoor activity among school-age children. The insufficient rate of outdoor activity among Beijing school-age children (< 2 h/day) was 82.9%, while the adequate rate was 17.1% (≥2 h/day). Differences in the insufficient outdoor activity rate among students defined by residential area, gender, grade, number of children in family, caregivers' daily outdoor activity duration, and beverage consumption before, during, and after outdoor activity were statistically significant (all *p* < 0.05).

**Table 2:** Univariate analysis of the prevalence of insufficient outdoor activity among school-age children in Beijing, China.

Characteristics	Daily outdoor activity, *n* (%)		χ^2^	*P*-value
	Insufficient (<2 h/day)	Sufficient (≥2 h/day)		
Total	2,795 (82.9)	575 (17.1)		
Residential area				
Central urban	1,014 (86.7)	156 (13.3)	17.626	< 0.001
Outer urban	870 (80.9)	206 (19.2)		
Suburban	911 (81.0)	213 (19.0)		
Gender				
Boys	1,361 (79.8)	344 (20.2)	23.642	< 0.001
Girls	1,434 (86.1)	231 (13.9)		
Grade				
Primary school	915 (87.2)	134 (12.8)	70.470	< 0.001
Middle school	884 (75.5)	287 (24.5)		
High school	996 (86.6)	154 (13.4)		
Ethnicity				
Han	2,554 (82.9)	527 (17.1)	0.046	0.830
Others	241 (83.4)	48 (16.6)		
Household registration type				
Agricultural	332 (84.1)	61 (15.9)	0.394	0.530
Urban	2,473 (82.8)	514 (17.2)		
Weight type status				
Normal	1,542 (82.8)	321 (17.2)	6.039	0.110
Underweight	188 (87.9)	26 (12.1)		
Overweight	487 (80.8)	116 (19.2)		
Obese	578 (83.8)	112 (16.2)		
Beverage consumption before outdoor activities				
Nothing	481 (86.2)	77 (13.8)	33.711	< 0.001
Only plain water	1,467 (83.7)	286 (16.3)		
Occasional beverage	655 (81.7)	147 (18.3)		
Sometimes beverage	160 (79.6)	41 (20.4)		
Often beverage	32 (57.1)	24 (42.9)		
Beverage consumption during outdoor activities				
Nothing	692 (84.0)	132 (16.0)	30.077	< 0.001
Only plain water	1,255 (84.4)	232 (15.6)		
Occasional beverage	565 (83.7)	110 (16.3)		
Sometimes beverage	227 (75.7)	73 (24.3)		
Often beverage	56 (66.7)	28 (33.3)		
Beverage consumption after outdoor activitiess				
Nothing	197 (81.7)	44 (18.3)	31.063	< 0.001
Only plain water	1,339 (85.5)	227 (14.5)		
Occasional beverage	835 (83.3)	167 (16.7)		
Sometimes beverage	331 (76.8)	100 (23.2)		
Often beverage	93 (71.5)	37 (28.5)		
≥2	1,068 (81.0)	251 (19.0)	5.927	0.015
1	1,727 (84.2)	324 (15.8)		
Caregiver's education				
High school or below	1028 (81.7)	230 (18.3)	2.114	0.146
College or above	1767 (83.7)	345 (16.3)		
**Caregivers' daily outdoor activity duration**				
< 0.5 h/day	665 (87.7)	93 (12.3)	74.889	< 0.001
0.5–1 h/day	1,220 (84.3)	227 (15.7)		
1–2 h/day	664 (82.9)	137 (17.1)		
≥2 h/day	246 (67.6)	118 (32.4)		

### 3.4 Multivariable binary logistic regression analysis of insufficient outdoor activity

#### 3.4.1 Overall adjusted associations

As shown in Table 3, multivariable analysis identified several independent factors associated with insufficient outdoor activity. Compared with children living in suburban areas, those residing in central urban areas exhibited significantly higher odds of insufficient outdoor activity (OR = 1.477, 95% CI: 1.152–1.893). No significant difference was observed between outer urban and suburban areas. Girls had higher odds of insufficient outdoor activity relative to boys (OR = 1.607, 95% CI: 1.317–1.962). Compared with middle school students, both primary school (OR = 2.010, 95% CI: 1.583–2.552) and high school students (OR = 2.109, 95% CI: 1.683–2.643) showed significantly elevated odds. For weight status, only obese children had higher odds of insufficient outdoor activity than children with normal weight (OR = 1.311, 95% CI: 1.020–1.686). No significant associations were found for underweight or overweight status. Regarding beverage consumption before outdoor activity, frequent beverage intake was associated with lower odds of insufficient outdoor activity compared with consuming nothing (OR = 0.461, 95% CI: 0.219–0.970). No significant associations were detected for beverage consumption during or after outdoor activity. Shorter daily outdoor activity duration among caregivers was strongly and independently associated with higher odds of insufficient outdoor activity in children. Relative to caregivers with ≥2 h per day, children whose caregivers spent < 0.5 h (OR = 3.150, 95% CI: 2.285–4.344), 0.5–1 h (OR = 2.326, 95% CI: 1.767–3.06), and 1–2 h (OR = 2.082, 95% CI: 1.546–2.805) outdoors daily all showed substantially higher odds. Ethnicity, household registration type, number of children in the family, and caregiver educational level were not significantly associated with insufficient outdoor activity.

**Table 3:** Multivariate binary logistic regression analysis of insufficient outdoor activity among school-aged children.

Independent variable	*B*	*S.E*	Wald χ^2^	*OR* (95% *CI*)	*P*-value
Residential area (ref: suburban)					
Central urban	0.390	0.127	9.495	1.477 (1.152–1.893)	0.002
Outer urban	0.009	0.115	0.007	1.010 (0.806–1.264)	0.934
Gender (ref: boys)					
Girls	0.474	0.102	21.760	1.607 (1.317–1.962)	< 0.001
Grade (ref: middle school)					
Primary school	0.698	0.122	32.769	2.010 (1.583–2.552)	< 0.001
High school	0.746	0.115	42.005	2.109 (1.683–2.643)	< 0.001
Ethnicity (ref: han)					
Others	−0.038	0.172	0.050	0.962 (0.687–1.347)	0.823
Household registration type (ref: agricultural)					
Urban	0.155	0.161	0.925	1.167 (0.852–1.599)	0.336
Weight type status (ref: normal)					
Underweight	0.370	0.226	2.687	1.447 (0.930–2.252)	0.101
Overweight	0.005	0.127	0.001	1.005 (0.784–1.288)	0.970
Obese	0.271	0.128	4.466	1.311 (1.020–1.686)	0.035
Beverage consumption before outdoor activities (ref: nothing)					
Only plain water	−0.274	0.156	3.055	0.761 (0.560–1.034)	0.080
Occasional beverage	−0.218	0.173	1.584	0.804 (0.573–1.129)	0.208
Sometimes beverage	−0.057	0.243	0.055	0.944 (0.586–1.521)	0.814
Often beverage	−0.774	0.379	4.159	0.461 (0.219–0.970)	0.041
Beverage consumption during outdoor activities (ref: nothing)					
Only plain water	−0.033	0.136	0.058	0.968 (0.742–1.262)	0.810
Occasional beverage	0.013	0.163	0.006	1.013 (0.736–1.394)	0.936
Sometimes beverage	−0.287	0.191	2.256	0.751 (0.516–1.091)	0.133
Often beverage	−0.309	0.339	0.830	0.735 (0.378–1.427)	0.362
Beverage consumption after outdoor activities (ref: nothing)					
Only plain water	0.263	0.200	1.733	1.301 (0.879–1.924)	0.188
Occasional beverage	0.132	0.208	0.403	1.141 (0.759–1.715)	0.526
Sometimes beverage	−0.177	0.226	0.613	0.838 (0.538–1.305)	0.434
Often beverage	−0.097	0.324	0.090	0.907 (0.481–1.711)	0.764
Number of children in famliy (ref: ≥2)					
1	0.185	0.099	3.504	1.203 (0.991–1.459)	0.061
Caregiver's education (ref: high school or below)					
College or above	−0.032	0.105	0.095	0.968 (0.789–1.189)	0.758
Caregivers' daily outdoor activity duration (ref: ≥2 h/day)					
< 0.5 h/day	1.148	0.164	49.013	3.150 (2.285–4.344)	< 0.001
0.5–1 h/day	0.844	0.140	36.307	2.326 (1.767–3.06)	< 0.001
1–2 h/day	0.733	0.152	23.271	2.082 (1.546–2.805)	< 0.001

#### 3.4.2 Stratified analysis by residential area

Table 4 presents results from multivariable binary logistic regression analyses stratified by residential area (central urban, outer urban, and suburban areas). In central urban area, compared with middle school students, primary school (OR = 3.711, 95% CI: 2.302–5.984) and high school students (OR = 3.507, 95% CI: 2.248–5.472) had substantially higher odds of insufficient outdoor activity. No significant associations were observed for gender, weight status, beverage consumption, ethnicity, household registration, family structure, caregiver education, or caregiver outdoor activity duration (all *P* > 0.05).

**Table 4:** Multivariate binary logistic regression analysis of insufficient outdoor activity among school-aged children, stratified by residential area.

Independent variable	Central urban areas		Outer urban areas		Suburban areas	
	*OR* (95% *CI)*	*P*	*OR* (95% *CI)*	*P*	*OR* (95% *CI*)	*P*
Gender (ref: boys)						
Girls	1.316 (0.896–1.931)	0.161	2.322 (1.632–3.306)	< 0.001	1.351 (0.974–1.874)	0.072
Grade (ref: middle school)						
Primary school	3.711 (2.302–5.984)	< 0.001	1.975 (1.291–3.021)	0.002	1.209 (0.809–1.807)	0.354
High school	3.507 (2.248–5.472)	< 0.001	1.710 (1.157–2.527)	0.007	1.750 (1.188–2.580)	0.005
Ethnicity (ref: han)						
Others	0.997 (0.576–1.724)	0.991	1.139 (0.598–2.169)	0.692	0.860 (0.468–1.580)	0.628
Household registration type (ref: agricultural)						
Urban	2.307 (0.260–20.460)	0.453	0.912 (0.563–1.477)	0.709	1.413 (0.900–2.219)	0.134
Weight type status (ref: normal)						
Underweight	0.895 (0.422–1.896)	0.772	2.161 (1.009–4.627)	0.047	1.422 (0.614–3.295)	0.411
Overweight	0.734 (0.462–1.166)	0.190	1.269 (0.817–1.969)	0.289	1.009 (0.667–1.528)	0.966
Obese	1.039 (0.632–1.708)	0.881	2.446 (1.543–3.878)	< 0.001	0.943 (0.634–1.403)	0.771
Beverage consumption before outdoor activities (ref: nothing)						
Only plain water	0.692 (0.375–1.277)	0.239	0.922 (0.543–1.564)	0.763	0.633 (0.377–1.061)	0.083
Occasional beverage	0.753 (0.388–1.462)	0.402	0.844 (0.467–1.526)	0.575	0.725 (0.409–1.285)	0.271
Sometimes beverage	1.355 (0.485–3.784)	0.563	0.947 (0.424–2.113)	0.894	0.654 (0.295–1.451)	0.296
Often beverage	1.245 (0.176–8.822)	0.826	0.265 (0.072–0.982)	0.047	0.185 (0.053–0.65)	0.008
Beverage consumption during outdoor activities (ref: nothing)						
Only plain water	0.990 (0.594–1.650)	0.970	1.203 (0.765–1.891)	0.425	0.774 (0.492–1.217)	0.267
Occasional beverage	1.302 (0.707–2.398)	0.397	1.542 (0.880–2.703)	0.130	0.535 (0.313–0.913)	0.022
Sometimes beverage	0.970 (0.440–2.137)	0.939	0.908 (0.474–1.740)	0.771	0.599 (0.327–1.096)	0.096
Often beverage	0.362 (0.124–1.060)	0.064	3.359 (0.806–13.998)	0.096	0.471 (0.143–1.554)	0.216
Beverage consumption after outdoor activities (ref: nothing)						
Only plain water	1.830 (0.750–4.466)	0.184	1.034 (0.513–2.081)	0.926	1.154 (0.640–2.079)	0.633
Occasional beverage	1.255 (0.509–3.092)	0.622	1.049 (0.509–2.163)	0.898	0.985 (0.528–1.837)	0.961
Sometimes beverage	0.833 (0.320–2.168)	0.708	0.774 (0.347–1.723)	0.530	0.749 (0.377–1.487)	0.409
Often beverage	0.570 (0.179–1.814)	0.341	0.721 (0.219–2.381)	0.592	1.581 (0.489–5.119)	0.444
Number of children in family (ref: ≥2)						
1	1.163 (0.789–1.715)	0.445	1.278 (0.913–1.788)	0.153	1.064 (0.776–1.460)	0.700
Caregiver's education (ref: high school or below)						
College or above	0.692 (0.426–1.125)	0.138	1.173 (0.837–1.643)	0.355	0.925 (0.660–1.295)	0.649
Caregivers' daily outdoor activity duration (ref: ≥2 h/day)						
< 0.5 h/day	1.596 (0.802–3.176)	0.183	6.368 (3.588–11.301)	< 0.001	2.722 (1.612–4.597)	< 0.001
0.5–1 h/day	1.429 (0.765–2.671)	0.263	3.314 (2.099–5.230)	< 0.001	2.247 (1.427–3.539)	< 0.001
1–2 h/day	1.646 (0.819–3.309)	0.162	2.578 (1.574–4.222)	< 0.001	1.923 (1.197–3.089)	0.007

In outer urban area, females had higher odds than males (OR = 2.322, 95% CI: 1.632–3.306); primary school (OR = 1.975, 95% CI: 1.291–3.021) and high school students (OR = 1.710, 95% CI: 1.157–2.527) had higher odds than middle schoolers; obese children had higher odds than normal-weight peers (OR = 2.446, 95% CI: 1.543–3.878); and underweight children also had higher odds (OR = 2.161, 95% CI: 1.009–4.627). Frequent beverage consumption before outdoor activity was associated with lower odds (OR = 0.265, 95% CI: 0.072–0.982). Relative to caregivers with ≥2 h/day of outdoor activity, children whose caregivers spent < 0.5 h (OR = 6.368, 95% CI: 3.588–11.301), 0.5–1 h (OR = 3.314, 95% CI: 2.099–5.230), and 1–2 h (OR = 2.578, 95% CI: 1.574–4.222) outdoors daily had markedly higher odds of insufficient outdoor activity.

In suburban area, High school students (OR = 1.750, 95% CI: 1.188–2.580) had higher odds than middle school students. Regarding beverage consumption, occasional consumption during outdoor activity (OR = 0.535, 95% CI: 0.313–0.913) was associated with lower odds, and frequent consumption before outdoor activity (OR = 0.185, 95% CI: 0.053–0.650) was also associated with lower odds. Caregiver outdoor activity duration remained a significant factor: children with caregivers spending < 0.5 h (OR = 2.722, 95% CI: 1.612–4.597), 0.5–1 h (OR = 2.247, 95% CI: 1.427–3.539), and 1–2 h (OR = 1.923, 95% CI: 1.197–3.089) outdoors daily had higher odds of insufficient outdoor activity.

#### 3.4.3 Stratified analysis by gender

Table 5 presents results from multivariable binary logistic regression analyses stratified by gender, to explore gender-specific patterns of associations with insufficient outdoor activity. Among boys, central urban residence was associated with higher odds of insufficient outdoor activity compared with suburban residence (OR = 1.399, 95% CI: 1.010–1.938), while no significant difference was observed between outer urban and suburban areas. Relative to middle school boys, primary school (OR = 1.912, 95% CI: 1.376–2.658) and high school boys (OR = 1.718, 95% CI: 1.279–2.308) had significantly higher odds. Underweight boys had higher odds than normal-weight boys (OR = 1.802, 95% CI: 1.003–3.237), while overweight and obese status showed no significant associations. Frequent beverage consumption before outdoor activity was associated with lower odds (OR = 0.394, 95% CI: 0.165–0.939). Shorter caregiver outdoor activity duration was strongly associated with higher odds of insufficient outdoor activity. Relative to caregivers with ≥2 h/day, boys whose caregivers spent < 0.5 h (OR = 2.885, 95% CI: 1.880–4.429), 0.5–1 h (OR = 2.084, 95% CI: 1.437–3.023), and 1–2 h (OR = 1.777, 95% CI: 1.190–2.652) outdoors daily had elevated odds. Ethnicity, household registration type, number of children in the family, caregiver education, and beverage consumption during/after outdoor activity were not significantly associated (all *P* > 0.05).

**Table 5:** Multivariate binary logistic regression analysis of insufficient outdoor activity among school-aged children, stratified by gender.

Independent variable	Boys		Girls	
	*OR* (95% *CI)*	*P*	*OR* (95% *CI*)	*P*
Residential area (ref: suburban)				
Central urban	1.399 (1.010–1.938)	0.044	1.618 (1.095–2.391)	0.016
Outer urban	0.906 (0.669–1.226)	0.522	1.169 (0.828–1.649)	0.375
Grade (ref: middle school)				
Primary school	1.912 (1.376–2.658)	< 0.001	2.049 (1.441–2.913)	< 0.001
High school	1.718 (1.279–2.308)	< 0.001	2.903 (1.998–4.218)	< 0.001
Ethnicity (ref: han)				
Others	1.289 (0.802–2.072)	0.294	0.692 (0.425–1.126)	0.138
Household registration type (ref: agricultural)				
Urban	1.154 (0.747–1.781)	0.519	1.169 (0.731–1.87)	0.515
Weight type status (ref: normal)				
Underweight	1.802 (1.003–3.237)	0.049	0.982 (0.500–1.930)	0.958
Overweight	0.886 (0.644–1.220)	0.459	1.256 (0.825–1.911)	0.288
Obese	1.337 (0.985–1.815)	0.062	1.377 (0.862–2.198)	0.181
Beverage consumption before outdoor activities (ref: nothing)				
Only plain water	0.665 (0.428–1.035)	0.071	0.804 (0.52–1.245)	0.328
Occasional beverage	0.700 (0.439–1.116)	0.134	0.889 (0.531–1.489)	0.655
Sometimes beverage	0.907 (0.497–1.657)	0.751	0.947 (0.392–2.286)	0.904
Often beverage	0.394 (0.165–0.939)	0.036	0.52 (0.099–2.723)	0.439
Beverage consumption during outdoor activities (ref: nothing)				
Only plain water	1.127 (0.778–1.633)	0.526	0.772 (0.521–1.142)	0.195
Occasional beverage	1.350 (0.876–2.080)	0.174	0.707 (0.433–1.156)	0.167
Sometimes beverage	0.901 (0.566–1.434)	0.66	0.563 (0.286–1.106)	0.095
Often beverage	0.825 (0.397–1.716)	0.607	0.971 (0.135–6.997)	0.976
Beverage consumption after outdoor activities (ref: nothing)				
Only plain water	1.608 (0.935–2.766)	0.086	1.091 (0.609–1.955)	0.769
Occasional beverage	1.027 (0.594–1.776)	0.923	1.403 (0.748–2.633)	0.291
Sometimes beverage	0.748 (0.421–1.330)	0.323	1.184 (0.562–2.495)	0.657
Often beverage	0.898 (0.414–1.948)	0.785	1.054 (0.303–3.667)	0.934
Number of children in family (ref: ≥2)				
1	1.189 (0.915–1.546)	0.195	1.320 (0.982–1.774)	0.066
Caregiver's education (ref: high school or below)				
College or above	1.110 (0.846–1.457)	0.450	0.797 (0.577–1.100)	0.167
Caregivers' daily outdoor activity duration (ref: ≥2 h/day)				
< 0.5 h/day	2.885 (1.880–4.429)	< 0.001	3.772 (2.300–6.186)	< 0.001
0.5–1 h/day	2.084 (1.437–3.023)	< 0.001	2.739 (1.811–4.142)	< 0.001
1–2 h/day	1.777 (1.190–2.652)	0.005	2.744 (1.739–4.329)	< 0.001

Among girls, central urban residence was associated with higher odds of insufficient outdoor activity compared with suburban residence (OR = 1.618, 95% CI: 1.095–2.391), with no significant difference between outer urban and suburban areas. Relative to middle school girls, primary school (OR = 2.049, 95% CI: 1.441–2.913) and high school girls (OR = 2.903, 95% CI: 1.998–4.218) had substantially higher odds. Caregiver outdoor activity duration remained a strong predictor: girls whose caregivers spent < 0.5 h (OR = 3.772, 95% CI: 2.300–6.186), 0.5–1 h (OR = 2.739, 95% CI: 1.811–4.142), and 1–2 h (OR = 2.744, 95% CI: 1.739–4.329) outdoors daily had significantly higher odds of insufficient outdoor activity. No significant associations were observed for weight status, beverage consumption (before, during, or after outdoor activity), ethnicity, household registration type, number of children in the family, or caregiver education (*P* > 0.05).

#### 3.4.4 Stratified analysis by grade

Table 6 presents results from grade-stratified multivariable binary logistic regression analyses, to explore grade-specific associations with insufficient outdoor activity. Among primary school students, compared with suburban area, students in central urban area had a higher odds of insufficient outdoor activity (OR = 2.594, 95% CI: 1.516–4.439). Compared with caregivers' outdoor duration ≥2 h/day, students whose caregivers spent < 0.5 h/day (OR = 4.745, 95% CI: 2.456–9.165), 0.5–1 h/day (OR = 3.693, 95% CI: 2.096–6.506), and 1–2 h/day (OR = 3.274, 95% CI: 1.786–5.998) outdoors daily had markedly higher odds.

**Table 6:** Multivariate binary logistic regression analysis of insufficient outdoor activity among school-aged children, stratified by grade.

Independent variable	Primary school		Middle school		High school	
	*OR* (95% *CI*)	*P*	*OR* (95% *CI*)	*P*	*OR* (95% *CI*)	*P*
Residential area (ref: suburban)						
Central urban	2.594 (1.516–4.439)	0.001	1.006 (0.706–1.434)	0.974	1.770 (1.063–2.949)	0.028
Outer urban	1.210 (0.761–1.924)	0.420	0.955 (0.678–1.346)	0.794	0.833 (0.543–1.277)	0.402
Gender (ref: girls)						
Boys	1.300 (0.878–1.923)	0.190	1.519 (1.129–2.044)	0.006	2.381 (1.572–3.605)	< 0.001
Ethnicity (ref: han)						
Others	1.316 (0.597–2.904)	0.496	0.815 (0.496–1.339)	0.419	0.967 (0.529–1.767)	0.913
Household registration type (ref: agricultural)						
Urban	1.241 (0.652–2.363)	0.510	0.931 (0.554–1.566)	0.788	1.522 (0.890–2.603)	0.125
Weight type status (ref: normal)						
Underweight	1.399(0.696–2.812)	0.346	1.38(0.657–2.901)	0.395	1.376(0.508–3.732)	0.530
Overweight	1.115 (0.613–2.026)	0.721	1.150 (0.797–1.657)	0.455	0.749 (0.483–1.161)	0.197
Obese	1.274 (0.758–2.142)	0.361	1.597 (1.095–2.33)	0.015	1.012 (0.631–1.623)	0.962
Beverage consumption before outdoor activities (ref: nothing)						
Only plain water	0.567 (0.320–1.007)	0.053	0.840 (0.538–1.310)	0.442	0.828 (0.404–1.698)	0.607
Occasional beverage	0.843 (0.399–1.784)	0.656	1.053 (0.650–1.707)	0.835	0.557 (0.268–1.159)	0.118
Sometimes beverage	0.988 (0.237–4.116)	0.986	1.181 (0.596–2.342)	0.633	0.675 (0.277–1.647)	0.388
Often beverage	1.639 (0.084–32.024)	0.745	0.359 (0.133–0.972)	0.044	0.490 (0.117–2.059)	0.330
Beverage consumption during outdoor activities (ref: nothing)						
Only plain water	1.357 (0.793–2.322)	0.265	0.916 (0.628–1.336)	0.649	0.669 (0.377–1.186)	0.169
Occasional beverage	0.811 (0.407–1.617)	0.551	0.951 (0.608–1.487)	0.825	1.227 (0.636–2.368)	0.543
Sometimes beverage	0.493 (0.229–1.058)	0.069	0.769 (0.44–1.343)	0.355	1.051 (0.506–2.185)	0.894
Often beverage	0.182 (0.006–5.405)	0.325	0.844 (0.322–2.214)	0.730	0.577 (0.206–1.62)	0.296
Beverage consumption after outdoor activities (ref: nothing)						
Only plain water	1.58 (0.813–3.073)	0.177	1.263 (0.701–2.274)	0.437	1.116 (0.414–3.012)	0.828
Occasional beverage	2.151 (1.000–4.625)	0.050	1.043 (0.569–1.911)	0.891	0.723 (0.271–1.932)	0.518
Sometimes beverage	1.442 (0.569–3.659)	0.441	0.867 (0.450–1.671)	0.670	0.461 (0.168–1.266)	0.133
Often beverage	8.359 (0.379–184.539)	0.179	0.957 (0.371–2.471)	0.928	0.406 (0.121–1.365)	0.145
Number of children (ref: ≥2)						
1	1.059 (0.713–1.574)	0.776	1.184 (0.894–1.569)	0.238	1.318 (0.897–1.938)	0.160
Caregiver's education (ref: high school or below)						
College or above	0.661 (0.413–1.058)	0.084	0.993 (0.736–1.339)	0.962	1.290 (0.876–1.901)	0.198
Caregivers' daily outdoor activity duration (ref: ≥2 h/day)						
< 0.5 h/day	4.745 (2.456–9.165)	< 0.001	3.497 (2.176–5.621)	< 0.001	2.044 (1.102–3.788)	0.023
0.5–1 h/day	3.693 (2.096–6.506)	< 0.001	2.184 (1.467–3.250)	< 0.001	1.791 (1.036–3.097)	0.037
1–2 h/day	3.274 (1.786–5.998)	< 0.001	2.277 (1.462–3.548)	< 0.001	1.271 (0.715–2.260)	0.414

Among middle school students, boys (OR = 1.519, 95% CI: 1.129–2.044) had higher odds of insufficient outdoor activity than girls. Obese middle school students (OR = 1.597, 95% CI: 1.095–2.330) had higher odds than normal weight peers, while underweight and overweight status showed no significant associations. Frequent beverage consumption before outdoor activity was associated with lower odds (OR = 0.359, 95% CI: 0.133–0.972). Relative to caregivers with ≥2 h/day, students with caregivers spending < 0.5 h (OR = 3.497, 95% CI: 2.176–5.621), 0.5–1 h (OR = 2.184, 95% CI: 1.467–3.250), and 1–2 h (OR = 2.277, 95% CI: 1.462–3.548) outdoors daily had higher odds.

Among high school students, central urban residence was associated with higher odds of insufficient outdoor activity compared with suburban residence (OR = 1.770, 95% CI: 1.063–2.949), while outer urban residence showed no significant difference. Boys had substantially higher odds than girls (OR = 2.381, 95% CI: 1.572–3.605), representing the strongest gender effect among all grade groups. Compared with caregivers' daily outdoor duration ≥2 h/day, students whose caregivers spent < 0.5 h/day (OR = 2.044, 95% CI: 1.102–3.788) and 0.5–1 h/day outdoors (OR = 1.791, 95% CI: 1.036–3.097) had higher odds of insufficient daily outdoor duration.

## 4 Discussion

This study found a high prevalence of inadequate outdoor activity among school-age children in Beijing, China. Over 80% of the children surveyed did not meet the recommended daily outdoor activity time of at least 2 h. A 2019 national survey on the physical fitness and health of Chinese students reported that 95.2% of school-age children had insufficient outdoor activity (< 2 h/day), with 83.26% having severe insufficiency (< 1 h/day) (28). At the regional level, the prevalence of insufficient outdoor activity exceeded 90% in Beijing, Tianjin, Shanghai, Guangdong, and Sichuan (28). In the present 2024 survey, 83.0% of children failed to meet the 2 h threshold, and 47.4% had < 1 h/day. These results are lower than the 2019 national average of 95.2% but higher than the prevalence rates reported in Beijing's local surveys in 2019 (71.9%) and 2022 (65.0%) (17, 28), and are generally consistent with existing regional and national findings. The widespread insufficient outdoor activity in these study highlights a major public health challenge for Chinese school-aged children. With rapid technological, economic, and educational development, children worldwide, including those in China, spend decreasing time outdoors. Interconnected factors likely contribute to this pattern: excessive screen time, heavy academic pressure occupying daily schedules, and greater reliance on motorized transport, all of which reduce opportunities for outdoor activity (15, 29). These contextual factors help explain the high prevalence observed in Beijing children and underscore the need for targeted strategies to address underlying behavioral and environmental constraints.

In this study, children in urban areas showed a higher prevalence of insufficient outdoor activity than those in suburban areas, consistent with previous reports (30, 31). Potential explanations include higher urban population density, lower per capita green space, greater motorized transport use, and increased traffic safety concerns, which may limit outdoor activity access (32, 33). In line with prior studies (15, 28, 31), girls were more likely to have insufficient outdoor activity than boys. This pattern may reflect physiological tendencies and traditional socialization patterns that lead girls to prefer indoor activities relative to boys (16, 34). We also observed higher insufficiency among primary and high school students than middle school students. High school students may face intense academic pressure that limits time outdoors; many sacrifice breaks for studying, and limited outdoor time may compound risks from prolonged near-work and sitting (35, 36). Middle school students, by contrast, may maintain more outdoor activity due to physical education requirements linked to the high school entrance examination (35, 36). These patterns suggest that intervention efforts may prioritize urban residents, girls, and primary and high school students.

In the full sample, obese children showed the highest prevalence of insufficient outdoor activity. In subgroup analyses, elevated prevalence among obese children was most evident in outer urban area and among middle schoolers, consistent with earlier findings (37). One plausible interpretation is that children with obesity may be less likely to engage in outdoor activity, and lower outdoor activity may reduce energy expenditure, creating a reciprocal pattern linked to excess weight gain (38). In the outer urban subgroup, underweight children also showed higher insufficiency. Given growing concerns about childhood obesity, these patterns highlight the need for public health and education authorities to consider outdoor activity support for children across weight statuses.

This study found relatively high proportions of beverage consumption before, during, and after outdoor activity (30.5%, 31.4%, and 46.3%, respectively). In the full sample, children who often consumed beverages before outdoor activity showed lower prevalence of insufficient outdoor activity. Similar patterns appeared in suburban boys and middle schoolers. Among primary school children, those who drank only plain water before outdoor activity also had lower prevalence. In suburban children, occasional beverage use during activity was associated with lower insufficiency, as was occasional use among primary schoolers. High beverage intake around outdoor activity likely reflects physiological needs, taste preferences, peer influences, and environmental availability, consistent with behavioral patterns in this age group (39, 40). Previous studies note that children frequently prefer sugar-sweetened and carbonated drinks (39, 40). Because childhood is a critical developmental period, such patterns may be associated with health risks including dehydration, metabolic disturbances, and dental problems (41, 42). These observations highlight the need for heightened awareness among caregivers, educators, and public health authorities regarding potential health risks associated with unregulated beverage intake during outdoor activity. Future behavioral interventions may prioritize age-appropriate guidance to support healthy beverage choices aligned with children's actual physical activity requirements.

Consistent with previous research (42, 43), the present study identified a robust cross-sectional association between caregiver behavior and children's outdoor activity. This association was consistent across most subgroups, except central urban students. Specifically, shorter daily outdoor activity duration among caregivers was strongly associated with higher likelihood of insufficient outdoor activity among children. These observational associations are consistent with the hypothesis that caregivers may serve as important role models and social facilitators for children's outdoor participation. Given these cross-sectional findings, public health programs may consider caregivers' focused education to raise awareness of the links between adult and child outdoor activity patterns. Encouraging caregivers to engage in regular outdoor activity may help create a supportive home environment that promotes children's outdoor time, which could support short and long term health. Such implications are consistent with the observed associations and should be interpreted as hypothesis generating rather than evidence of confirmed causal pathways.

This study has several limitations that should be acknowledged. First, the cross-sectional baseline design precludes causal interpretations of observed associations, particularly the temporal relationship between caregivers' outdoor activity and children's outdoor activity participation. Reverse causality and unobserved confounding cannot be ruled out. Second, all data on outdoor activity duration and beverage consumption were self-reported, which may introduce recall bias and social desirability bias. Although we used a standardized 1-week recall period and fixed response categories to reduce misclassification, self-reported estimates remain less reliable than device-measured physical activity. Third, participants were recruited from only three urban districts in Beijing, which may restrict the generalizability of our findings to rural children, other geographic regions in China, or children with chronic health conditions. Fourth, we did not collect data on certain potential confounders, including neighborhood built environment, access to green spaces, screen time, sleep patterns, family socioeconomic status, and school physical education policies, which may independently affect children's outdoor activity. Fifth, caregiver outdoor activity was assessed only as daily duration without capturing activity type, intensity, or whether caregivers engaged in activities together with children, limiting our understanding of the underlying mechanisms. Despite these limitations, this large scale population based study provides robust evidence on the prevalence and correlates of insufficient outdoor activity among urban school-aged children in Beijing, with a particular focus on caregiver-related factors, offering valuable implications for future intervention design.

## 5 Conclusion

In conclusion, this cross-sectional study reveals a high prevalence of insufficient outdoor activity among school-aged children in Beijing, with more than 80% failing to meet the daily recommended duration. Shorter caregiver outdoor activity time, urban area, girls, primary or high school, and obesity are significantly associated with a higher likelihood of insufficient outdoor activity. Frequent beverage consumption before outdoor activity shows a negative association with activity insufficiency, while beverage intake during or after activity shows no consistent relationships. These observational findings support the notion that caregiver outdoor activity is closely linked to children's outdoor activity patterns, and highlight important variations across residential areas, gender, and grade groups. Given the cross-sectional design, these results should be interpreted as correlational rather than causal, and no firm conclusions on directionality or mechanism can be drawn. This study underscores insufficient outdoor activity as a critical public health issue for urban school-aged children in China. Future prospective and longitudinal studies with objective activity measurements, multilevel confounding adjustment, and cluster-robust statistical methods are needed to clarify causal pathways. Intervention strategies that involve caregivers and address sociodemographic disparities may be meaningful targets for future public health efforts to improve outdoor activity levels in this population.

## Data Availability

The original contributions presented in the study are included in the article/Supplementary material, further inquiries can be directed to the corresponding authors.

## References

[1] Chen D Wang J Chen J Lu M Du Y Zhu Z . Smartwatch-monitored physical activity and myopia in children: a 2-year prospective cohort study. BMC Med. (2025) 23:294. doi: 10.1186/s12916-025-04136-5 40399891 PMC12096555

[2] World Health Organization. Guidelines on Physical Activity and Sedentary Behaviour. (2020). Available online at: https://www.who.int/publications/b/55501 (Accessed February 14, 2026).

[3] Chinese Nutrition Society. Chinese Dietary Guidelines. Beijing: Chinese Nutrition Society (2022). 254–63 p.

[4] Zhou X Pardue MT Iuvone PM Qu J. Dopamine signaling and myopia development: what are the key challenges. Prog Retin Eye Res. (2017) 61:60–71. doi: 10.1016/j.preteyeres.2017.06.003 28602573 PMC5653403

[5] Rueter K Jones AP Siafarikas A Chivers P Prescott SL Palmer DJ . The influence of sunlight exposure and sun protecting behaviours on allergic outcomes in early childhood. Int J Environ Res Public Health. (2021) 18:5429. doi: 10.3390/ijerph18105429 34069576 PMC8161152

[6] Voortman T van den Hooven EH Heijboer AC Hofman A Jaddoe VW Franco OH . Vitamin D deficiency in school-age children is associated with sociodemographic and lifestyle factors. J Nutr. (2015) 145:791–8. doi: 10.3945/jn.114.208280 25833782

[7] Qin Z Liang G Hu Y Fu Y Yang Z Zhao Y . Relationship between outdoor activity, BMI, and vitamin D status in children. Chin J Child Health. (2017) 25:334–7. doi: 10.11852/zgetbjzz2017-25-04-04

[8] Liu WW Wu XY Tao FB. Promotion of outdoor activities on children's mental health and its potential mechanism. Chin J Sch Health. (2020)41:1277–80. doi: 10.16835/j.cnki.1000-9817.2020.08.044

[9] Sun ZY Liu ZH Feng BJ Zhang XW Xu K Chen L. Relationship between physical activity, sleep duration, overweight/obesity, and depressive symptoms among Tianjin middle school students. Chin J Sch Health. (2023) 44:489–93. doi: 10.16835/j.cnki.1000-9817.2023.04.003

[10] Duckham RL Procter-Gray E Hannan MT Leveille SG Lipsitz LA Li W. Sex differences in circumstances and consequences of outdoor and indoor falls in older adults in the MOBILIZE Boston cohort study. BMC Geriatr. (2013) 13:133. doi: 10.1186/1471-2318-13-133 24313971 PMC3907046

[11] Kido A Miyake M Watanabe N. Interventions to increase time spent outdoors for preventing incidence and progression of myopia in children. Cochrane Database Syst Rev. (2024) 6:CD013549. doi: 10.1002/14651858.CD013549.pub2 38864362 PMC11167692

[12] Zhu Z Chen Y Tan Z Xiong R McGuinness MB Müller A. Interventions recommended for myopia prevention and control among children and adolescents in China: a systematic review. Br J Ophthalmol. (2023)107:160–6. doi: 10.1136/bjophthalmol-2021-319306 34844916

[13] National health commission of the people's republic of China website. Guidelines for the Prevention and Control of Myopia. (2024). Available online at: https://www.gov.cn/zhengce/zhengceku/202406/content_6957665.htm (Accessed February 23, 2026).

[14] Clark R Kneepkens SCM Plotnikov D Shah RL Huang Y Tideman JWL . UK Biobank eye and vision consortium. Time spent outdoors partly accounts for the effect of education on myopia invest. Ophthalmol Vis Sci. (2023) 64:38. doi: 10.1167/iovs.64.14.38 PMC10683767 38010695

[15] Guthold R Stevens GA Riley LM Bull FC. Global trends in insufficient physical activity among adolescents: a pooled analysis of 298 population-based surveys with 1.6 million participants. Lancet Child Adolesc Health. (2020) 4:23–35. doi: 10.1016/S2352-4642(19)30323-2 31761562 PMC6919336

[16] Xu RB Gao D Wang ZH Zou ZY Hu PJ Jun M . Analysis of the current status of outdoor activity time among Chinese students in 2016. Chin J Child Health Care. (2018) 26:254–7. doi: 10.11852/zgetbjzz2018-26-03-07

[17] Wang L Zhao H Xia ZW Sun BJ Teng LX Guo X . Comparison of eye-related behaviors of Beijing primary and secondary school students in 2019 and 2022. Chin J Chronic Dis Prev Control. (2024) 32:527–29.

[18] Bassett DR John D Conger SA Fitzhugh EC Coe DP. Trends in physical activity and sedentary behaviors of United States youth. J Phys Act Health. (2015) 12:1102–11. doi: 10.1123/jpah.2014-0050 25347913

[19] Nigg C Weber C Schipperijn J Reichert M Oriwol D Worth A . Urban-Rural differences in children's and adolescent's physical activity and screen-time trends across 15 years. Health Educ Behav. (2022) 4:10901981221090153. doi: 10.1177/10901981221090153 35506637

[20] Yotsukura E Torii H Mori K Ogawa M Hanyuda A Negishi K . Slowing of greater axial length elongation stemming from the coronavirus disease 2019 pandemic with increasing time outdoors: the Tokyo myopia study. Ophthalmol Sci. (2024) 4:100491. doi: 10.1016/j.xops.2024.100491 38827490 PMC11141272

[21] Chinese Government Website. Communiqué of the Seventh National Population Census. (2021). Available online at: https://www.gov.cn/guoqing/2021-05/13/content_5606149.htm (Accessed March 24, 2026).

[22] UNICEF. Atlas of China's Child Development Indicators. (2024). Available online at: https://www.unicef.cn/media/28801/file/%E4%B8%AD%E5%9B%BD%E5%84%BF%E7%AB%A5%E5%8F%91%E5%B1%95%E6%8C%87%E6%A0%87%E5%9B%BE%E9%9B%862024.pdf (Accessed March 24, 2026).

[23] Beijing Municipal Commission of Education. Statistical Overview of Beijing's Education Development in the 2023-2024 Academic Year. (2024). Available online at: https://jw.beijing.gov.cn/xxgk/shujufab/tongjigaikuang/202403/t20240321_3596738.html (Accessed March 22, 2026).

[24] Yang Y Ge Y. Encyclopedia of Nutrition Science. Beijing: People's Medical Publishing House (2019). 1157–60 p.

[25] National Health Commission of the People's Republic of China. Anthropometric Measurements Method in Health Surveillance (WS/T 424-2013). (2013). Available online at: https://www.nhc.gov.cn/wjw/yingyang/201308/1f27caef0b22493e93a1da8aec2cd63a/files/1739783545878_39188.pdf (Accessed February 23, 2026).

[26] National Health Commission of the People's Republic of China. Screening Standard for Malnutrition of School-Age Children and Adolescents (WS/T 456-2014). (2014). Available online at: https://www.nhc.gov.cn/wjw/pqt/201407/38b15c0a1ed444e8908e12752decaffa/files/1739781146755_22583.pdf (Accessed February 23, 2026).

[27] National Health Commission of the People's Republic of China. Screening for Overweight and Obesity Among School-Age Children and Adolescents (WS/T 586-2018). (2018). Available online at: http://www.nhc.gov.cn/ewebeditor/uploadfile/2018/03/20180330094031236.pdf (Accessed February 23, 2026).

[28] Chen L Zhang Y Chen M Ma T Ma Q Liu JY . Prevalence of unhealthy lifestyle behaviors among Chinese Han children and adolescents. Chin J Cardiovasc Dis. (2022) 50:1177–85. doi: 10.3760/cma.j.cn112148-20220826-00648 36517438

[29] Marques A Henriques-Neto D Peralta M Martins J Demetriou Y Schönbach DMI . Prevalence of physical activity among adolescents from 105 low, middle, and high-income countries. Int J Environ Res Public Health. (2020) 17:3145. doi: 10.3390/ijerph17093145 32365969 PMC7246732

[30] Christiana RW Bouldin ED Battista RA. Active living environments mediate rural and non-rural differences in physical activity, active transportation, and screen time among adolescents. Prev Med Rep. (2021) 23:101422. doi: 10.1016/j.pmedr.2021.101422 34159049 PMC8193609

[31] Gao F Guo Q Wang B Cao S Qin N Zhao L . Distributions and determinants of time spent outdoors among school-age children in China. J Expo Sci Environ Epidemiol. (2022)32:223–31. doi: 10.1038/s41370-021-00401-w 34980893

[32] Manyanga T Barnes JD Chaput JP Katzmarzyk PT Prista A Tremblay MS . Prevalence and correlates of adherence to movement guidelines among urban and rural children in mozambique: a cross-sectional study. Int J Behav Nutr Phys Act. (2019) 16:94. doi: 10.1186/s12966-019-0861-y 31661004 PMC6819612

[33] Wang K Liu J. The spatiotemporal trend of city parks in mainland china between 1981 and 2014: implications for the promotion of leisure time physical activity and planning. Int J Environ Res Public Health. (2017) 14:1150. doi: 10.3390/ijerph14101150 28961182 PMC5664651

[34] Lisowski P Kantanista A Bronikowski M. Are There any differences between first grade boys and girls in physical fitness, physical activity, BMI, and sedentary behavior? Results of HCSC study. Int J Environ Res Public Health. (2020) 17:1109. doi: 10.3390/ijerph17031109 32050548 PMC7038200

[35] Xu F Wang L Li F Wang X Liu J Wang Y . Association between eyesight and outdoor activity in primary and middle school students in Henan Province. Chin J Health Educ. (2023) 39:601–4. doi: 10.16168/j.cnki.issn.1002-9982.2023.07.005

[36] Jin JX. An intervention study on the effect of increased outdoor activity time on the incidence and progression of myopia among primary and secondary school students. Hefei, AH: Anhui Medical University (2015).

[37] Dong LX Yang Y Ma QQ Huang Y Zhang SJ Li J. Effects of video and outdoor time on screening myopia combined with overweight/obesity in children and adolescents. South China J Prev Med. (2024) 50:241–45. doi: 10.12183/j.scjpm.2024.0241

[38] Sun ZY Liu ZH Feng BJ Zhang XW Xu K Chen L. Relationship between physical activity, sleep duration and overweight, obesity with depressive symptoms among midle school students in Tianjin. Chin J Sch Health. (2023) 44:489–93. doi: 10.16835/j.cnki.1000-9817.2023.04.003

[39] Watts AW Miller J Larson NI Eisenberg ME Story MT Neumark-Sztainer D . Multicontextual correlates of adolescent sugar-sweetened beverage intake. Eat Behav. (2018) 30:42–8. doi: 10.1016/j.eatbeh.2018.04.003 29777969 PMC6314180

[40] Li Y Meng L Zhang Y Li W Liang S Zheng J . Association between sugar-sweetened beverage consumption and childhood overweight and obesity: a cross-sectional study in Beijing, China. Front Nutr. (2025) 12:1686320. doi: 10.3389/fnut.2025.1686320 41256920 PMC12620238

[41] Deng J Liu T Long Z. Factors affecting outdoor physical activity (OPA) in children and adolescents: a systematic review and meta-analysis. Heliyon. (2024) 10:e38859. doi: 10.1016/j.heliyon.2024.e38859 39524901 PMC11543882

[42] Ostrin LA Sajjadi A Benoit JS. Objectively measured light exposure during school and summer in children. Optom Vis Sci. (2018) 95:332–42. doi: 10.1097/OPX.0000000000001208 29554008 PMC5880726

[43] Cohen A Lang JJ Prince SA Colley RC Tremblay MS Chaput JP. Are adolescents who do physical activity with their parents more active and mentally healthier? Health Rep. (2025) 36:19–33. doi: 10.25318/82-003-x202500100002-eng 39832193

