# Supplementary material for: Prevalence of insufficient outdoor activity and caregiver-related correlates among school-aged children in Beijing, China: a cross-sectional analysis
Source: Front Public Health. 2026 May 26;14:1816425. doi: 10.3389/fpubh.2026.1816425 (PMC13246481; doi:10.3389/fpubh.2026.1816425)
Supplement: Supplementary file 1 [file Table_1.doc]

Supplementary Table 1 BMI cutoff points for screening overweight and obesity by sex and age in school-aged children and adolescents aged 6 - 18 years(Kg/m²)

| Age(years) | Boys | | Girls | |
| --- | --- | --- | --- | --- |
|  | Overweight | Obesity | Overweight | Obesity |
| 6.0～ | 16.4 | 17.7 | 16.2 | 17.5 |
| 6.5～ | 16.7 | 18.1 | 16.5 | 18.0 |
| 7.0～ | 17.0 | 18.7 | 16.8 | 18.5 |
| 7.5～ | 17.4 | 19.2 | 17.2 | 19.0 |
| 8.0～ | 17.8 | 19.7 | 17.6 | 19.4 |
| 8.5～ | 18.1 | 20.3 | 18.1 | 19.9 |
| 9.0～ | 18.5 | 20.8 | 18.5 | 20.4 |
| 9.5～ | 18.9 | 21.4 | 19.0 | 21.0 |
| 10.0～ | 19.2 | 21.9 | 19.5 | 21.5 |
| 10.5～ | 19.6 | 22.5 | 20.0 | 22.1 |
| 11.0～ | 19.9 | 23.0 | 20.5 | 22.7 |
| 11.5～ | 20.3 | 23.6 | 21.1 | 23.3 |
| 12.0～ | 20.7 | 24.1 | 21.5 | 23.9 |
| 12.5～ | 21.0 | 24.7 | 21.9 | 24.5 |
| 13.0～ | 21.4 | 25.2 | 22.2 | 25.0 |
| 13.5～ | 21.9 | 25.7 | 22.6 | 25.6 |
| 14.0～ | 22.3 | 26.1 | 22.8 | 25.9 |
| 14.5～ | 22.6 | 26.4 | 23.0 | 26.3 |
| 15.0～ | 22.9 | 26.6 | 23.2 | 26.6 |
| 15.5～ | 23.1 | 26.9 | 23.4 | 26.9 |
| 16.0～ | 23.3 | 27.1 | 23.6 | 27.1 |
| 16.5～ | 23.5 | 27.4 | 23.7 | 27.4 |
| 17.0～ | 23.7 | 27.6 | 23.8 | 27.6 |
| 17.5～ | 23.8 | 27.8 | 23.9 | 27.8 |
| 18.0～ | 24.0 | 28.0 | 24.0 | 28.0 |

The data is from the standard titled Screening for Overweight and Obesity among School-age Children and Adolescents (WS/T 586-2018)，which issued by the National Health Commission of the People's Republic of China(27).
